# Challenges faced by medical officers in managing snakebite envenoming in North Karnataka, India: A qualitative thematic analysis

**DOI:** 10.4314/ahs.v26i1.5

**Published:** 2026-03

**Authors:** J Dodda Basava, N Chandan, Veerappa A Kothiwale, Phaniraj Vastrad, Kalesh M Karun, Manish J Barvaliya

**Affiliations:** 1 ICMR–National Institute of Traditional Medicine, Belagavi, Karnataka, India; 2 KLE Academy of Higher Education and Research (KAHER), Belagavi, Karnataka, India; 3 Model Rural Health Research Unit, Sirwar, Raichur, Karnataka, India

**Keywords:** Snakebite envenoming, antivenom, rural health, medical officers, qualitative research

## Abstract

**Background:**

Snakebite envenoming is a neglected tropical disease and a major medical emergency in rural India. Medical officers play a key role in its management but often face systemic and operational barriers.

**Objective:**

To explore the challenges and barriers faced by medical officers in managing snakebite envenoming in rural Karnataka.

**Methods:**

A qualitative study using purposive sampling was conducted in selected Government healthcare centers (PHCs (Primary healthcare centre), CHCs (Community health centre), and THs (Taluk hospital) across Belagavi and Raichur districts of Karnataka. The interviews were transcribed verbatim and analyzed using reflexive thematic analysis. Ethical clearance was obtained from the institutional ethics committees.

**Results:**

Forty-eight medical officers (38 males and 10 females) participated in open-ended, face-to-face interviews conducted in English and 41.66% had 1–5 years of experience. Major barriers included delayed patient presentation due to reliance on traditional healers, poor awareness, shortages of antivenom (ASV), equipment, ICU facilities, inadequate training, staffing shortages, and persistent cultural misconceptions about snakebite.

**Conclusion:**

Strengthening infrastructure, ensuring ASV availability, enhancing training, and community education are crucial to improve outcomes and reduce mortality from snakebite envenoming in rural India.

## Introduction

Snakebite is a major public health concern, affecting approximately 5.4 million individuals annually, resulting in up to 2.7 million cases of envenoming. Within this context, India remains the most heavily affected country, witnessing an estimated 1.2 million fatalities due to snakebites between 2000 and 2019, averaging 58,000 deaths per year^1^. The population suffering from snake bites varies regarding their socio-economic status, occupational backgrounds (including farmers, plantation labourers, herdsmen, fishermen, workers in snake-related industries, and other food producers), and residential settings such as rural or urban areas and living conditions^2^. Predominantly, snakebite incidents occur in rural and tribal areas characterized by limited transportation, infrastructure, particularly exacerbated during nighttime, thus increasing mortality rates^3^. The underlying reasons for this high mortality rate attributed to snakebites encompass various factors such as the insufficiency of antivenom (ASV), challenges related to prompt access to healthcare facilities, inadequacies in health services, and reliance on traditional remedies^4^. Recognizing the public health burden of snakebite envenoming, the World Health Organization (WHO) classified it as a high-priority neglected tropical disease (NTD) in 2017. In May 2018, the Seventy-first World Health Assembly (WHA) adopted a resolution directing WHO to implement global strategies to reduce snakebite-related morbidity and mortality. Building on this, WHO released a strategic roadmap in 2019 aiming to halve deaths and disabilities from snakebite envenoming by 2030. In India, where snakebite remains a major rural health problem, these global initiatives are aligned with the National Action Plan for Snakebite Envenoming (NAPSE), which seeks to strengthen healthcare infrastructure, ensure consistent antivenom availability, and enhance the training of medical officers in effective snakebite management^5^-^7^.

Despite the publication of standard treatment guidelines (STGs) for snakebite management in 2017, the actual implementation of these protocols remains notably deficient within both public and private healthcare systems across India^8^. Consequently, the knowledge and adherence of healthcare workers (HCWs) to standardized procedures for snakebite diagnosis and treatment emerge as pivotal in effectively addressing snakebite cases^9^.

Moreover, healthcare workers often lack formal training regarding the proper handling of snakebite incidents, such as providing first aid, identification of snake varieties, administration of antivenom, and continuous monitoring of patients throughout their treatment^10^. Hence, this study aimed to explore the challenges and barriers faced by medical officers in the management of snakebite envenoming.

## Methodology

### Study setting

The study was carried out in the selected Government healthcare centres in Belagavi and Raichur districts of Karnataka state. In Belagavi, 9 taluks were selected, and in Raichur district, 3 taluks were selected based on snake bite burden. 27 health care centres were selected from Belagavi, and 9 health care centres were selected from Raichur district.

### Participants

Recruitment involved purposive sampling of medical officers currently working in Primary Health Centres (PHCs), Community Health Centres (CHCs) and taluk hospitals in the two districts. Inclusion criteria were medical officers actively involved in snakebite case management with varying years of experience. Those not directly managing snakebite cases or unwilling to consent were excluded. Of approximately 55 invited medical officers, 48 consented to participate (response rate 87%), with seven declining due to scheduling conflicts.

Interviews were conducted in the preferred language of the participant (English), depending on comfort and fluency, to optimize communication and data quality.

### Data Collection Procedures

Data were collected through semi-structured, face-to-face interviews using an interview guide developed specifically for the study. The guide covered key domains: perceived challenges in snakebite management, barriers encountered at the facility and community level, experiences with antivenom use, and recommendations for improvement. Before the main study, the guide was pilot-tested with six medical officers from healthcare centers not included in the final sample. Feedback from the pilot facilitated refinement of questions to improve clarity and relevance. Interviews were conducted in English, according to participant preference and comfort level. All interviews took place within the healthcare facility, most commonly in a private room to ensure confidentiality and minimize interruptions. Each interview lasted between 20–30 minutes, was audio-recorded with participant consent, and supplemented by detailed field notes documenting interviewer observations, participant nonverbal cues, and environmental context.

Transcripts were not returned to participants for comment or correction; however, participants were encouraged to clarify or elaborate on any points immediately during the interview. All interviews were conducted in person to allow rapport-building and nuanced exploration.

### Researcher team and reflexivity

The interviews were conducted by two authors, one female public health researcher and one male clinician, both with formal training and prior experience in qualitative methods. To further enhance reflexivity, researchers maintained a positionality log, reflecting on potential biases and expectations before, during, and after interviews. Both interviewers were aware of their professional roles and acknowledged the risk of social desirability bias, given their prior clinical experience in snakebite management.

To mitigate this, interviewers made clear to participants that there were no right or wrong answers, emphasized confidentiality, and actively invited honest discussion of challenges (including negative experiences or criticisms of the health system). Interviewers avoided leading questions and refrained from sharing their own management practices or opinions during data collection. The research team also included analysts who had not participated in data collection to provide an external perspective during coding and theme review.

### Connection with Participants

There was no prior relationship between the interviewers and participants. Before each interview, researchers introduced themselves and explained the study's purpose and their roles, ensuring participants were fully informed.

### Saturation

Data saturation was reached after 24 interviews when no new themes or insights emerged from subsequent interviews. Saturation here is defined as the point at which additional data collection no longer generated novel information relevant to the research questions, consistent with established qualitative research principles. The judgment of saturation was made jointly by two independent researchers engaged in coding and thematic analysis, who continuously reviewed emerging themes after each interview and compared findings to previous data. Despite reaching saturation early, interview sessions continued until 48 participants were interviewed to ensure diverse representation across facility types and experience levels.

### Data Analysis

All interview recordings were transcribed verbatim, ensuring precise capture of participant responses and contextual detail. Thematic analysis was conducted following the reflexive approach outlined by Braun and Clarke which is well-suited to qualitative health research and emphasizes rigor, transparency, and reflexivity in theme development^11^.

Coding procedures were primarily inductive, allowing themes to emerge naturally from the data rather than imposing predefined categories. Two researchers independently reviewed the transcripts, generating initial codes based on recurring concepts and experiences described by participants. The coding was single-blind at first, followed by collaborative discussions to resolve discrepancies and achieve consensus. A preliminary codebook was developed after coding five transcripts, guiding the continued analysis and allowing for refinement as new data emerged.

Codes representing related concepts were then clustered into subthemes (e.g., “late presentation,” “patient misconceptions”), which in turn were organized into broader themes (e.g., “Patient Presentation Issues”). This process was iterative and reflexive, ensuring careful consideration of context and diversity of experiences across settings, genders, and years of experience. No qualitative data analysis software was used; coding and theme consolidation were performed manually in Microsoft Excel.

Frequency counts of codes were used only to identify recurring challenges and assess saturation; these counts are not presented in a quasi-quantitative manner within the results to avoid misinterpretation. Instead, illustrative quotes from diverse participants (indicating district, gender, and years of experience) are provided for each major theme to highlight typical and variant experiences.

Theme refinement and final categorization were conducted by both analysts, with feedback from the broader research team including clinicians with snakebite management expertise to enhance credibility and validity.

## Ethical Considerations

The study protocol was reviewed and approved by the Institutional Human Ethics Committees of MRHRU, Sirwar, and KAHER, Belagavi. Ethical approval reference numbers are provided in the ethics approval letters (available upon request). The study was conducted in accordance with the Indian Council of Medical Research (ICMR) 2017 guidelines for ethical research. Prior to data collection, all participants received detailed information sheets in plain language and provided written informed consent for participation and audio recording. To ensure confidentiality, no identifying personal information was collected.

## Findings

### Participants characteristics

A total of 48 medical officers were included in the study. 37 medical officers were recruited using purposive sampling from the Belagavi district, 29 males (78.40%) and 8 females (21.60%), with a mean age of 35.30 years (SD=8.30) and 11 medical officers were recruited from the Raichur district in those 9 males (81.80%) and 2 females (18.20%) with a mean age of 38.90 years (SD=8.40). (Table 1)

**Table 1:** Basic details of medical officers

District	Frequency (%)
Belagavi	37(77.08)
Raichur	11(22.91)
**Gender**	
Male	38(79.20)
Female	10(20.80)
**Qualification**	
MBBS	20(41.66)
MBBS MD/MS/Diploma	28(58.33)
**Experience (In Years)**	
1-5	20(41.66)
5-10	10(20.83)
>10	18(37.51)

### Challenges and Barriers in Managing Snakebite Envenoming

Medical officers reported multifaceted challenges affecting effective snakebite management across different levels of care. These challenges stemmed from patient behaviour, healthcare system limitations, inadequate infrastructure, training gaps, shortages in essential drugs and equipment, and persistent community-level misconceptions.

### Patient Presentation Issues

Delayed reporting and patient misconceptions significantly hinder timely initiation of treatment. Many patients approach healthcare facilities late, often after first visiting traditional healers or attempting home remedies. Some refuse referral or testing, while others present with rapid deterioration or anxiety due to fear and misinformation.

*“Patients arrive after applying local herbs or cuts; by then, swelling and complications have already worsened.”* (PID-19, Female, 1 year experience)

*“Some believe the bite is non-poisonous and delay reaching a tertiary center.”* (PID-3, Female, 4 years experience)

*“Patients collapse within minutes; by the time they reach us, we can do very little.”* (PID-9, Male, 10 years experience)

Conversely, a few officers noted that early-reporting patients could be managed effectively:

*“When patients reach early and ASV is available, most cases recover well.”* (PID-38, Male, 20 years experience)

### Healthcare Facility Limitations

Inadequate infrastructure, limited ICU support, and the absence of continuous patient monitoring were major system-level barriers. Facilities at the periphery often lacked the capacity to manage complications, forcing repeated referrals. Poor ambulance connectivity further delayed critical care.

*“Our center lacks an ICU and trained staff for critical monitoring.”* (PID-17, Male, 5 years experience)

*“A separate snakebite cell and well-equipped taluk hospital are urgently needed.”* (PID-2, Male, 13 years experience)

*“Without proper ambulance and ventilator support, stabilization becomes difficult.”* (PID-20, Male, 12 years experience)

However, some health care centres reported relatively better arrangements:

*“Our facility maintains a functioning ambulance and adequate ASV stock, which helps in timely response.”* (PID-14, Female, 15 years experience)

### Training and Staffing Challenges

Lack of formal training in snakebite management and shortages of skilled personnel were among the most cited issues. Medical officers emphasized the need for structured training for physicians, nurses, and support staff.

*“Training is vital; most of us have learned by experience, not formal teaching.”* (PID-7, Male, 12 years experience)

*“Nursing officers are unsure about when and how to administer ASV.”* (PID-14, Female, 15 years)

*“Non-availability of physicians and support staff complicates monitoring, especially at night.”* (PID-12, Male, 22 years experience)

A few respondents noted that periodic district-level workshops improved confidence in handling cases:

*“Recent training sessions by district health officials helped us manage ASV reactions more confidently.”* (PID-31, Male, 10 years experience)

### Resource and Equipment Shortages

Shortages in anti-snake venom (ASV), diagnostic tests, and essential drugs were recurring problems. Laboratories lacked basic coagulation tests such as PT-INR, and stock-depletion of ASV sometimes interrupted treatment midway.

*“Deficiency in ASV and lab tests like INR slows down management.”* (PID-33, Male, 14 years)

*“Investigations come late; sometimes, we treat symptomatically before lab results arrive.”* (PID-39, Male, 10 years experience)

*“Deciding ASV dosage is difficult, especially when the type of snake is unknown.”* (PID-14, Female, 15 years experience)

In contrast, some officers acknowledged improved supply chains:

*“In recent months, we received regular ASV supplies, which reduced treatment delays.”* (PID-37, Male, 20 yearsexperience)

### Specific Case Management Issues

Complex cases, particularly paediatric and severe envenomation with complications like acute kidney injury (AKI)—posed significant management difficulties. Many centers lacked facilities for dialysis, intubation, or ventilator support. In Russell's viper bites, acute kidney injury (AKI) was recognized as a known complication requiring prompt referral to a district or tertiary care facility with dialysis services. This practice algns with the recommendations outlined in the Standard Treatment Guidelines^12^.

*“Handling AKI cases is challenging; dialysis is unavailable at our center.”* (PID-28, Male, 2 years experience)

*“Managing paediatric bites is different—they need continuous monitoring and gentler dosing.”* (PID-45, Female, 11 yearsexperience)

*“Even with experience, decisions about severe cases during night shift are difficult without a physician.”* (PID-24, Male, 3 years experience)

### Community and Cultural Barriers

Traditional healing practices, misconceptions, and panic-driven behaviours dominated community-level challenges. People frequently sought quacks or spiritual healers before visiting formal hospitals. Despite repeated awareness campaigns, adherence to scientific first-aid remained low.

*“Most villagers first go to traditional healers, wasting valuable time.”* (PID-8, Male, 15 years experience)

*“Convincing patients to accept ASV is difficult—they fear side effects.”* (PID-16, Female, 1 year experience)

*“Despite continuous IEC, they still apply herbal paste or cut the bite area.”* (PID-27, Male, 4 years experience)

Nevertheless, areas with consistent IEC activities saw better outcomes:

*“In our block, due to regular awareness sessions, more patients now report directly to PHCs.”* (PID-40, Male, 8 years experience)

### Opportunities for better management of snakebite cases

Improving snakebite management requires a focus on key areas, starting with enhancing public education and awareness. Misconceptions about venomous snakebites often lead to delayed medical care and reliance on traditional healers. Increasing public awareness and educating communities on the urgency of seeking professional medical treatment can help reduce these delays.

*“To improve the management of snakebite cases, addressing several key areas identified from the data is essential. Firstly, enhancing patient education and awareness is critical. Misconceptions about poisonous snakebites lead to delayed medical care and a tendency to seek alternative treatments from traditional healers or quacks.”* (PID-7, PID-13)

Healthcare facilities must be better equipped to handle snakebite emergencies.

Strengthening healthcare facilities is essential for effectively handling snakebite emergencies. This includes ensuring adequate resources such as antivenom, ICU facilities, and continuous patient monitoring systems. Addressing staffing shortages and providing specialized training for healthcare professionals can further improve care quality. Additionally, resolving issues like inadequate drug supply and limited access to laboratory tests is crucial for streamlining treatment.

Community barriers, such as reliance on traditional healers and a lack of awareness about modern treatment options, contribute to delayed reporting and care. Strengthening healthcare infrastructure and improving access to emergency services, including well-equipped ambulances, can enhance patient outcomes. Furthermore, focusing on specific case management aspects, such as snake identification and pediatric care, is vital for providing targeted and effective treatment. By addressing these areas, snakebite management can be significantly improved, leading to better patient outcomes and reduced mortality.

*“Focusing on specific case management issues, such as snake identification and paediatric care, is essential for providing targeted and effective treatment.”* (PID-4, PID-8)

**Figure 1:**
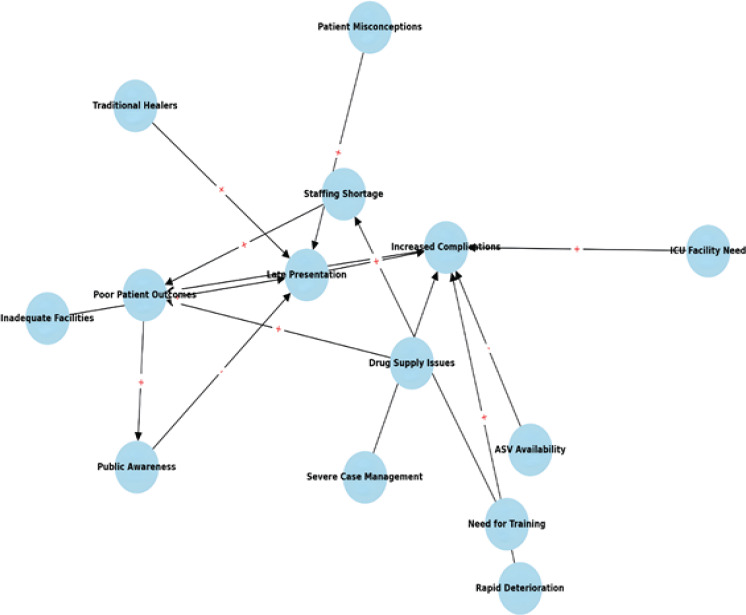
Causal Loop Diagram of Challenges and Barriers in Snakebite Management

A causal loop diagram (CLD) was developed based on recurring themes and causal statements from the interview data. It visually depicts how interconnected factors, such as delayed patient presentation, limited antivenom (ASV) availability, inadequate ICU facilities, and staffing shortages, reinforce one another, creating feedback loops that exacerbate challenges in snakebite management. The CLD helps illustrate the systemic nature of these barriers and highlights points where targeted interventions could improve patient outcomes. (Figure 1)

## Discussion

The findings from the present study conducted in Belagavi and Raichur districts of India are consistent with findings from several international studies on snakebite management, highlighting common barriers and opportunities across diverse contexts. A major theme that emerged from this study was the delayed presentation of snakebite victims, often due to cultural reliance on traditional healers and a general lack of awareness regarding the severity of snakebites. This challenge was similarly observed in Northern Uganda, where healthcare workers identified delayed treatment-seeking behaviour as a critical obstacle to effective care (Wafula et al., 2023)^10^. The preference for traditional medicine over formal healthcare, despite the potentially fatal nature of envenoming, indicates a widespread issue rooted in socio-cultural beliefs and misinformation that spans multiple regions.

Healthcare facility limitations were another dominant theme, with medical officers reporting shortages of essential equipment, ICU beds, ambulances, and trained staff in the Indian districts studied. These issues are consistent with findings from Sudan, where Badri et al. (2025) identified a weak healthcare system and limited access to antivenom as key barriers^13^. Similarly, in Kenya, health workers expressed concerns over poor infrastructure, insufficient antivenom stocks, and lack of emergency equipment (Barnes et al., 2021)^14^. These findings collectively highlight the systemic under-resourcing of rural and peripheral healthcare facilities as a critical challenge in snakebite management globally.

The shortage of trained staff was also highlighted in the present study, where medical officers acknowledged their limited expertise in administering ASV (Antivenom) and managing complications such as acute kidney injury (AKI). This aligns with findings from Uganda and Kenya, where healthcare workers reported a need for regular in-service training and updated clinical guidelines (Wafula et al., 2023; Barnes et al., 2021)^10^,^14^. Moreover, Gajbhiye et al. (2023) emphasized incorporating snakebite management into medical curricula and establishing model management centers to ensure a more competent and responsive workforce^8^.

Resource and antivenom shortages were frequently cited in both the Indian and international contexts. The Indian study participants noted the logistical and supply chain difficulties that led to inconsistent ASV availability, a sentiment echoed in the Sudanese and Ugandan contexts, where inadequate stocks of antivenom and diagnostic tools severely impaired treatment capacity (Badri et al., 2025; Wafula et al., 2023)^10^,^13^. Additionally, Gutiérrez et al., (2014) in Central America emphasized the urgent need for improved antivenom distribution networks, particularly in remote and underserved areas^16^.

Community and cultural barriers, such as mistrust of the healthcare system, preference for traditional practices, and low awareness of medical treatment, were consistently reported across the board. This study's insights from Belagavi and Raichur underscore the critical role of public education in addressing these issues, which is also a recurring recommendation in other studies. For instance, Wafula et al. (2023) and Gajbhiye et al. (2023) both advocated for mass education campaigns to correct misconceptions and promote timely care-seeking behaviour^8^,^10^.

Moreover, case complexity, especially involving pediatric patients or those with severe complications, emerged as a nuanced challenge in the Indian study. The inability to identify the snake species, combined with inadequate diagnostic facilities, was highlighted as a serious limitation. These concerns are mirrored in studies from Central America and Kenya, where case complexity and inadequate referral systems further exacerbated patient outcomes (Gutiérrez, 2014; Barnes et al., 2021)^14^,^16^.

Furthermore, previous studies, including (Bawaskar et al., 2014), have highlighted the need for diagnostic tools like the 20-minute whole blood clotting test (20WBCT), which are often unavailable in rural settings. This reflects reported practice, whereas the use of 20WBCT is recommended in the Standard Treatment Guidelines and the National Action Plan for Snakebite Envenoming (NAPSE)^6^,^12^,^17^.

On the opportunity front, the present study reinforces the global consensus that training, community education, and healthcare strengthening are key to better snakebite management. Consistent with the views of Gajbhiye et al. (2023), there is a need for national programs tailored to local epidemiology, including the establishment of region-specific venom banks and better compliance with WHO ASV production standards. Additionally, interdisciplinary collaboration, as suggested by Gutiérrez (2014) and Berg et al. (2024), could enhance resource allocation and research into alternative therapies^16^,^18^.

## Limitation

This study was conducted in only two districts (Belagavi and Raichur), which may limit the transferability of findings to other regions with differing healthcare systems or snakebite epidemiology. Only medical officers from public sector facilities were included, excluding perspectives from nurses, other healthcare staff, private practitioners, patients, and caregivers. Interviews were relatively short (20–30 minutes), potentially restricting the depth of insights. Responses may have been influenced by social desirability or courtesy bias, and the study did not incorporate triangulation methods such as facility audits, patient interviews, or member checking. Despite these limitations, the study provides important insights into the challenges faced by medical officers in managing snakebite envenoming in rural India.

## Recommendations

Improving snakebite care requires regular training for medical officers, stronger community awareness to reduce traditional healer reliance, and timely hospital visits. Ensuring steady ASV supply through efficient distribution and upgrading rural healthcare infrastructure, ICUs, ambulances, and medical resources will significantly enhance the healthcare system's capacity to manage snakebite cases effectively.

## Conclusion

This study highlights the significant challenges in snakebite management, including delays in seeking medical care, inadequate healthcare infrastructure, resource shortages, and cultural barriers. Addressing these issues requires a multifaceted approach involving medical training, community education, and healthcare improvements through capacity-building training programmes to ensure timely, appropriate, and effective treatment.

## Figures and Tables

**Table 2:** Themes, concise definitions, and representative quotes illustrating challenges and barriers faced by medical officers in snakebite management

Theme	Concise Definition	Representative Quotes
Patient Presentation Issues	Delay or avoidance of medical care due to misconceptions and reliance on traditional healers	“Patients arrive late after local treatments, worsening their condition.” (PID-19, F, 1 yr)
Healthcare Facility Limitations	Lack of ICU, monitoring systems, and emergency transport delays	“Without ICU and ambulance, stabilization becomes difficult.” (PID-20, M, 12 yr)
Training and Staffing Challenges	Insufficiently trained staff and a shortage of physicians, nurses, and technicians	“Nursing officers need more training on ASV administration.” (PID-14, F, 15 yr)
Resource and Equipment Shortages	Inadequate ASV, lab tests, drugs, or diagnostics	“Lack of ASV and lab support poses serious management challenges.” (PID-8, M, 15 yr)
Specific Case Management Issues	Difficulty managing severe, paediatric or AKI cases due to a lack of advanced care	“Dialysis and ventilator support are unavailable when needed.” (PID-28, M, 2 yr)
Community and Cultural Barriers	Traditional healer reliance, fear of ASV, and low awareness are delaying treatment	“People prefer traditional healers and reach the hospital late.” (PID-8, M, 15 yr)

## Data Availability

The data supporting the findings of this study are available from the corresponding author upon reasonable request.
